# 5-ALA fluorescence in a WHO grade I papillary glioneuronal tumour: a case report

**DOI:** 10.1007/s00701-020-04223-x

**Published:** 2020-01-27

**Authors:** José Pedro Lavrador, Hussein Shaaban Kandeel, Alison Kalb, Zita Reisz, Safa Al-Sarraj, Richard Gullan, Keyoumars Ashkan, Francesco Vergani, Ranjeev Bhangoo

**Affiliations:** King’s College Hospital NHS Foundation Trust, London, UK

**Keywords:** Glioneuronal, 5-Aminolevulinic acid, Fluorescence, WHO grade I

## Abstract

5-ALA is proven to be effective in high-grade glioma operative resection. The use of 5-ALA in WHO grade I lesions is still controversial. A 49-year-old lady was diagnosed in 2004 with a left temporal lobe lesion as an incidental finding; she was followed up clinically and radiologically. In 2016, the lesion showed contrast enhancement and she was offered surgical resection but given she is asymptomatic, she refused. In 2018, the lesion showed signs of transformation with ring contrast enhancement, increased vasogenic oedema and perfusion; the patient accepted surgery at that point. She had preoperative mapping by navigated transcranial magnetic stimulation and she had operative resection with 5-ALA. The tumour was bright fluorescent under Blue 400 filter—Zeiss Pentero 900©(Carl Zeiss Meditec)—and both bright fluorescence and pale fluorescence were resected. Postoperative MRI showed complete resection and histopathology revealed WHO grade I papillary glioneuronal tumour, negative for BRAF V600 mutation. WHO grade I papillary glioneuronal tumour may present as 5-ALA fluorescent lesions. From a clinical perspective, 5-ALA can be used to achieve complete resections in these lesions which, in most cases, can be curative.

**Electronic supplementary material:**

The online version of this article (10.1007/s00701-020-04223-x) contains supplementary material, which is available to authorized users.

## Background and importance

In the last decades, clinical and imagiological follow-up of asymptomatic low-grade lesions was common practice. In 2012, Jakola et al. [9] published one of the main studies responsible for a change in the treatment approach of these patients towards a safe and extensive surgical resection as the first treatment option. It was common practice to follow these patients until signs of transforming lesion were perceived in the imaging performed, as this strategy was not related with a decrease in quality of life or cognitive performance [1].

The current literature supports the continuous diametric expansion of these tumours [15] and the relevance of increased perfusion and contrast enhancement during the transforming process into higher grade lesions [14]. While still experimental and with inconsistent results in lower grade lesions [16], 5-ALA is an established adjuvant in the surgery of higher grade lesions [17] which renders this technique useful in supposed transforming low-grade lesions.

This conservative approach is rarely responsible for uncommon histological findings allowing for a better understanding of the natural history of rare lesions. In this report, the authors present a rare case of a clinical and imagiological natural history of a papillary glioneuronal tumour (PGNT) that was treated with a presumptive diagnosis of a transforming low-grade glioma/high-grade glioma.

## Clinical presentation

**Fig. 1:**
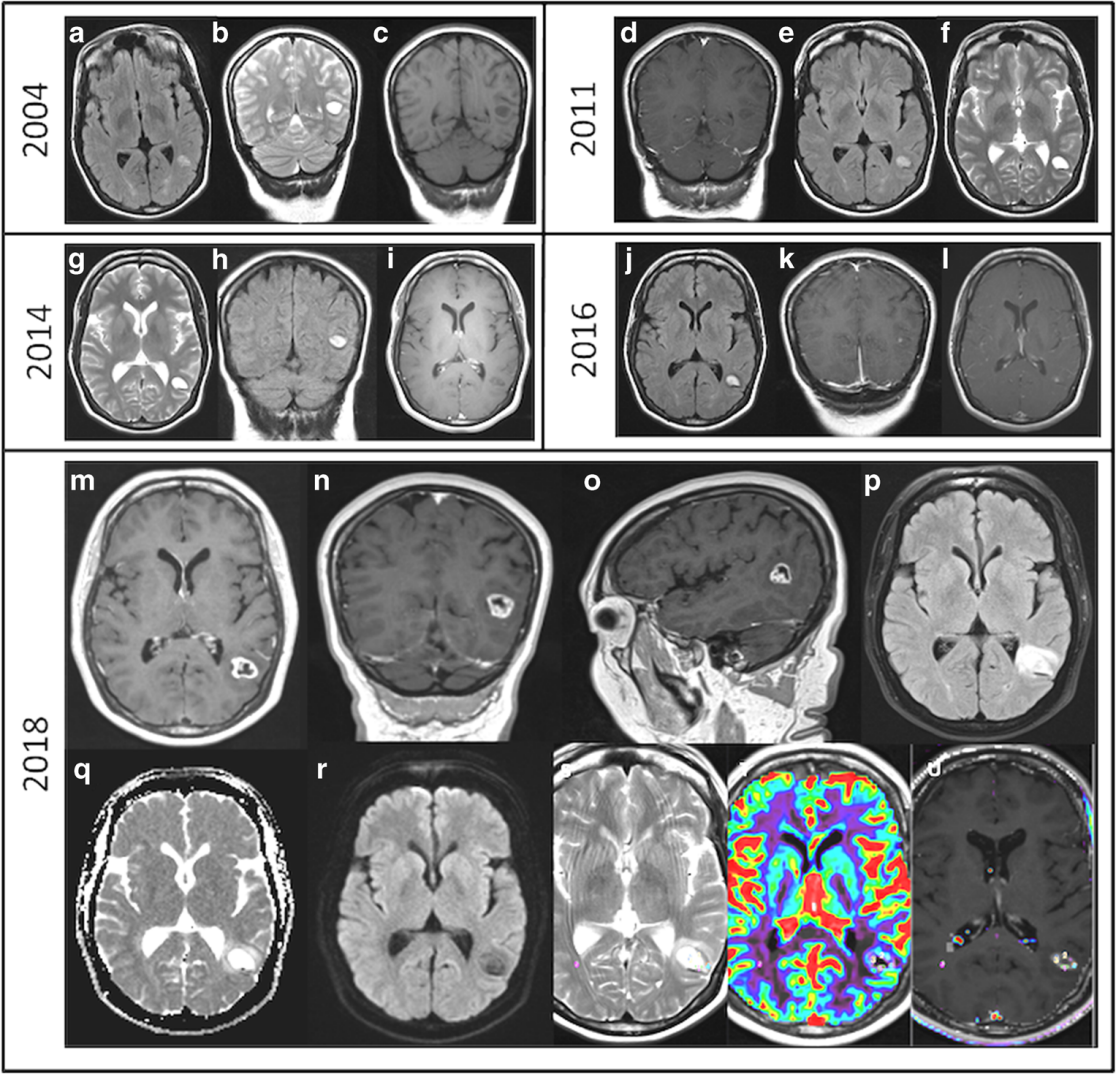
Evolution of the temporal-parietal-occipital lesion from 2004 to 2018. Slow growing lesion since 2004 with initial punctiform contrast uptake in 2016 that increased in size in 2018. 2004 (**a**, axial FLAIR; **b**, coronal T2; **c**, coronal T1); 2011 (**d**, coronal T1 GAD; **e**, axial FLAIR; **f**, axial T2); 2014 (**g**, axial T2; **h**, coronal FLAIR; **i**, axial T1 GAD); 2016 (**j**, axial FLAIR; **k**, coronal T1 GAD; **l**, axial T1 GAD); 2018 (**m**, axial T1 GAD; **n**, coronal T1 GAD; **o**, sagittal T1 GAD; **p**, axial FLAIR; **q**, ADC map; **r**, DWI; **s**, axial T2, **t**, **u**, CBV)

A 49-year-old right-handed bilingual lady (primary language Tamil, and secondary language English) was referred to our neuro-oncology department for follow-up of a lesion located in the posterior aspect of the left temporal lobe (temporo-occipital junction). She was incidentally diagnosed in 2004 (from image investigations for headaches) with a left temporal lobe lesion, and it was decided to monitor the lesion with regular clinical and imaging follow-up (Fig. 1a–c). The lesion remained stable until 2016 (Fig. 1d–i), when the lesion showed focal contrast enhancement (Fig. 1j–l). The patient continued to be asymptomatic, but as the lesion showed signs of progression, surgical treatment was offered, but was declined.

Two years later, she remained asymptomatic, but imaging of the lesion revealed further signs of transforming lesion with ring contrast enhancement, increased vasogenic oedema and perfusion (Fig. 1m–u). At this stage, the patient accepted the surgical treatment.

**Fig. 2:**
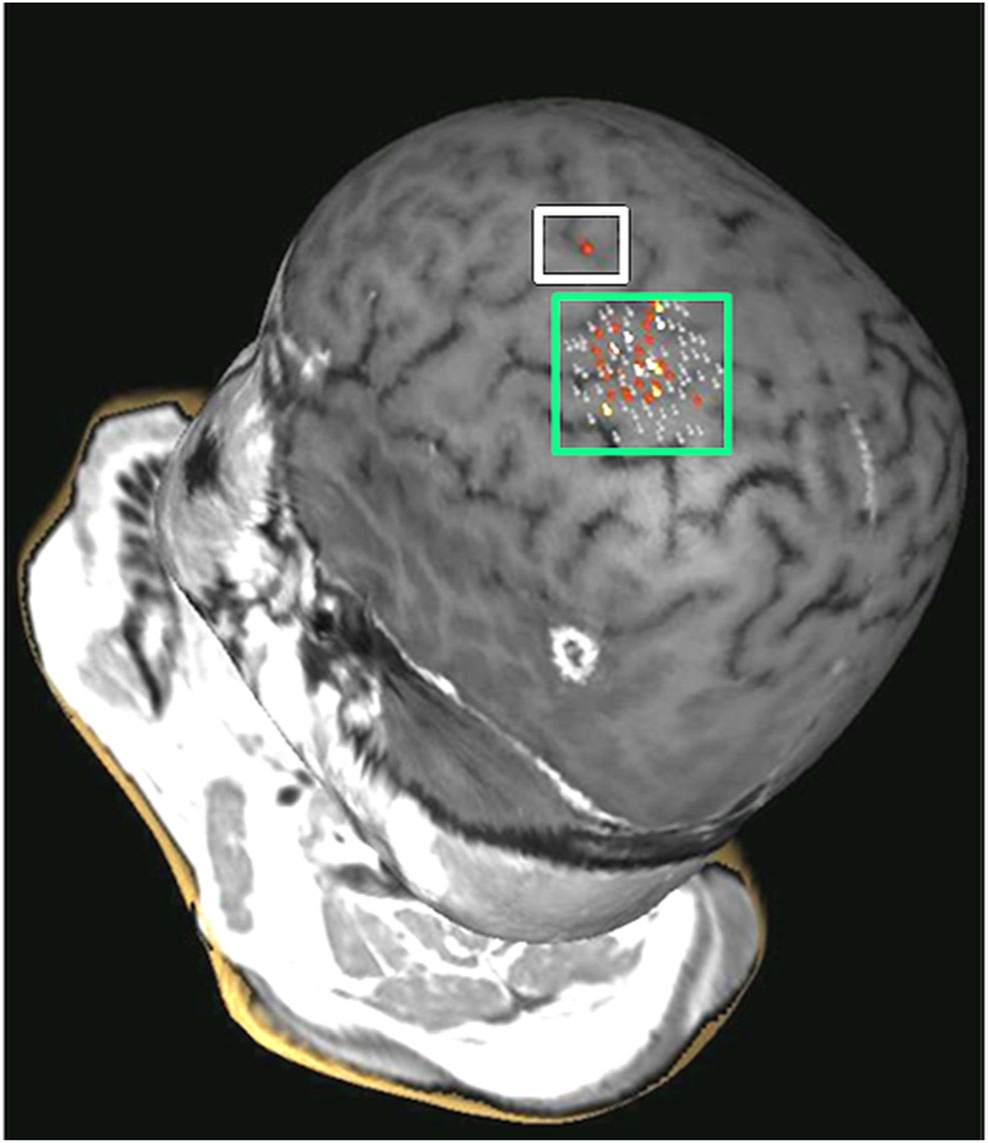
Preoperative navigated transcranial magnetic stimulation (nTMS). White box, positive responses for speech (arrest). Green box, positive responses in the hand muscles

**Fig. 3:**
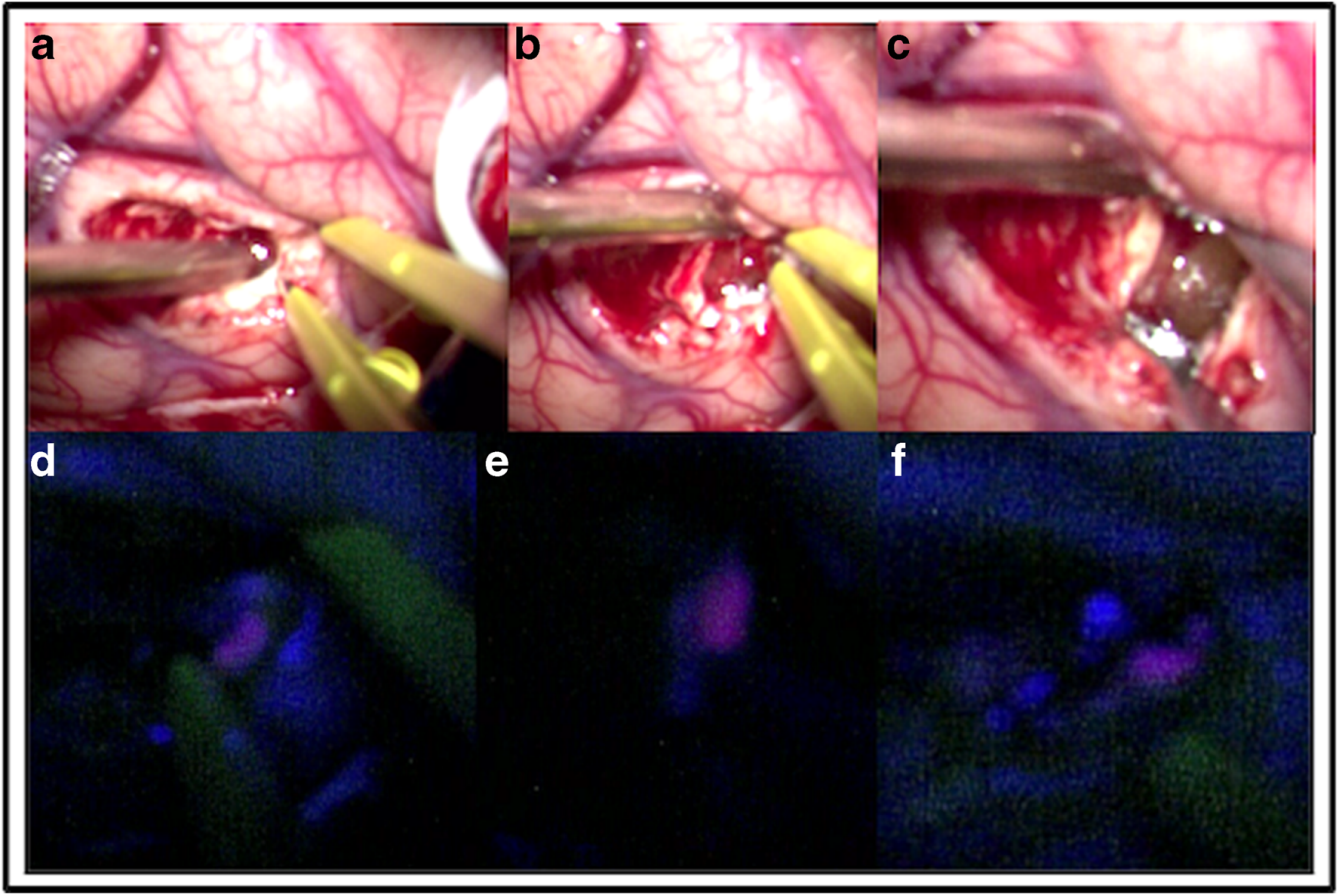
Intraoperative images. Correlation between the different moments of the surgery (white light versus 5-ALA). **a**, **d** Initial visualization of the tumour. **b**, **e** Initial dissection of the tumour from the surrounding white matter. **c**, **f** Exposure of the tumour

Preoperative brain mapping with navigated transcranial magnetic stimulation was performed—one site of speech arrest was found in the posterior frontal gyrus (Fig. 2, white square) in front of the area of the hand notch (Fig. 2, green square). She had surgery according to an asleep-awake-asleep protocol with bilingual negative mapping (no speech arrest, no anomia or comprehension deficit in both languages) and the use of 5-ALA (1500 mg was administrated 2 h before skin incision, oral route) as a surgical adjunct in tumour resection. The tumour was bright fluorescent under Blue 400 filter—Zeiss Pentero 900© (Carl Zeiss Meditec)—the gross tumour as no clear infiltration of the surrounding brain was perceived during the resection (Fig. 3, video). Both bright fluorescence and pale fluorescence were resected.

**Fig. 4:**
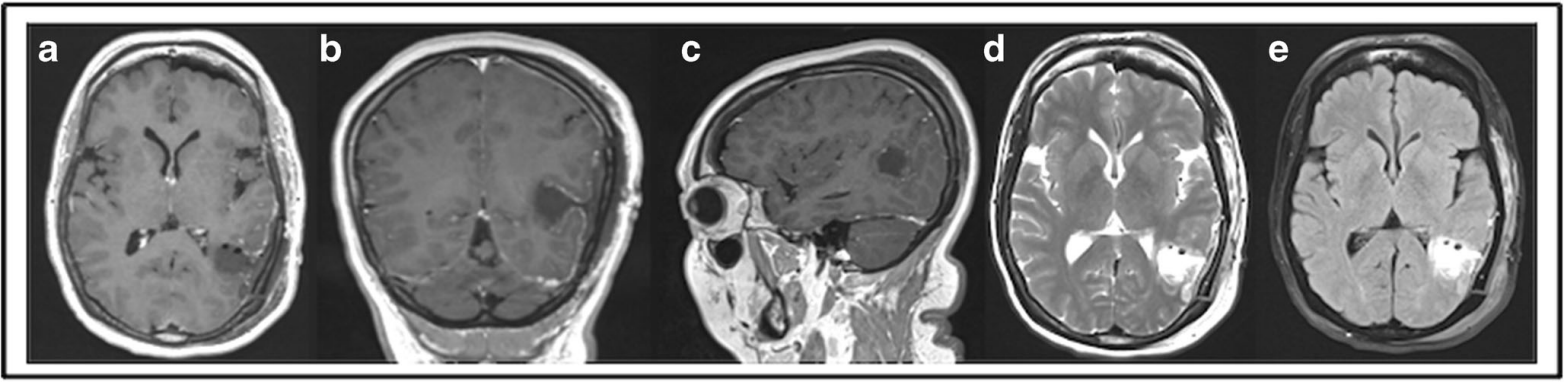
Postoperative MRI. Complete resection (gross total resection). **a** Axial T1 GAD. **b** Coronal T1 GAD. **c** Sagittal T1 GAD. **d** Axial T2. **e** Axial FLAIR

The patient experienced mild dysphasia postoperatively (naming difficulties, mainly in English) that recovered to her baseline after 2 weeks. The postoperative MRI documented completed resection (Fig. 4).

**Fig. 5:**
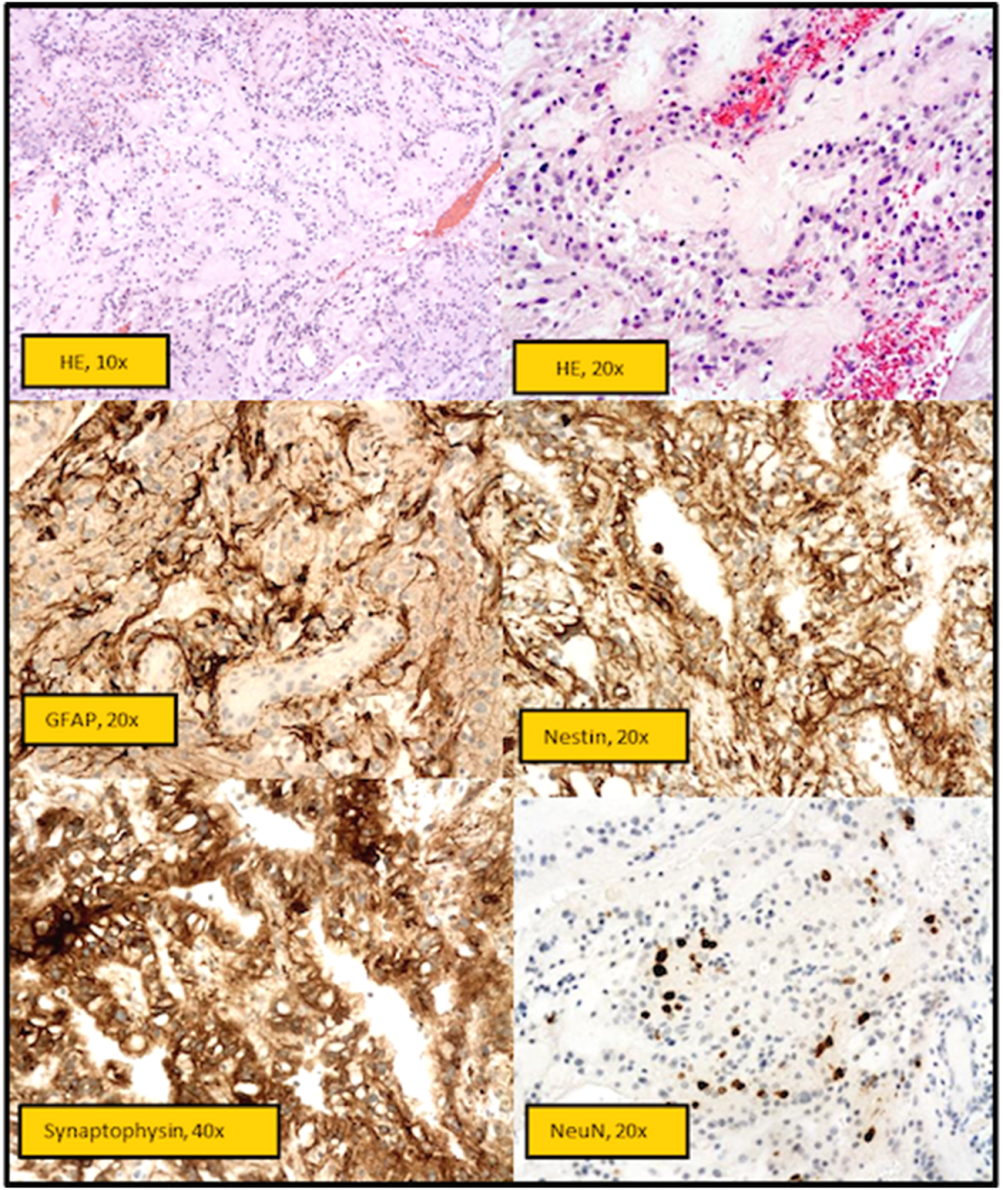
Histopathology. HEx10, moderately cellular biphasic tumour with a predominant pseudopapillary growth pattern and prominently hyalinised blood vessels. Hex20, the tumour is composed of a single layer of cuboidal or flattened glial cells around the hyalinised fibrovascular cores and intervening islands of relatively monomorphic neurocytic cells in fibrillary or slightly microcystic background. No significant cytological atypia, mitotic activity or necrosis is visible. GFAPx20, immunohistochemistry for GFAP highlights the astrocytic component mainly around the blood vessels. Nestinx20, the neoplastic cells are intensely expressing Nestin. Synaptophysinx40, the neuronal cells and the neuropil are strongly positive with synaptophysin, confirming the glioneuronal nature of the neoplastic process. NeuNx20, NeuN shows strong nuclear staining within the interpapillary neurocytic cells

The histopathological result revealed an unexpected WHO grade I papillary glioneuronal tumour, negative for BRAF V600 mutation (Fig. 5).

Given these diagnoses and the complete resection achieved with the surgical resection, the multidisciplinary team decision was to follow-up this patient with no adjuvant treatment.

## Discussion

WHO grade I papillary glioneuronal tumour is an uncommon diagnosis in the adult population. A recent review of the literature documented less than one hundred fifty cases reported in the literature [2]. The histogenesis of PGNT is controversial (multipotent precursor cells located in the subventricular zone capable of divergent glioneuronal differentiation, supported by its predominant periventricular location versus dedifferentiation from the secondary germinal layer) [4]. The scarce information about the natural history of these lesions support male gender, low cellular proliferation, and maximal surgical resection as positive prognostic indicators [2]. From an imaging perspective, the majority of these lesions present as a cystic lesion with a solid component [11], which was seen as well in this patient.

As far as the authors are aware, this is the first report of a 5-ALA fluorescent PGNT. Our group has reported other cases as well, of benign lesions that were fluorescent with 5-ALA [10]. Other cases of non-diffuse gliomas have been reported as well as potential target for 5-ALA-assisted resection, for example pilomyxoid [3] and pilocytic astrocytomas [5]. Goryaynov et al. [7] have reported a high incidence of 5-ALA fluorescence in WHO grade I tumours (5/5 patients diagnosed with WHO grade I tumours were found to have visible fluorescence—4 pilocytic astrocytomas and 1 desmoplastic infantile ganglioglioma).

When diffuse WHO grade II tumours are considered, the rates of 5-ALA-induced fluorescence found in the literature are quite heterogeneous (8% [6], 9% [20], 16% (the largest published series) [8], 33% [19], and 40% [12]) and therefore difficult to generalize. Goryaynov et al. [7] suggested a negative influence of the administration of anticonvulsants in the potential visible fluorescence in WHO grade II tumours (27% positive fluorescence on anticonvulsants versus 83% positive fluorescence with no anticonvulsants). This group suggested as well that 5-ALA can be particularly useful in detecting foci of early malignant transformation within the WHO grade II diffuse gliomas. However, this still requires further validation by larger studies.

The actual mechanism for 5-ALA uptake by the cells is not completely understood. Disturbances in the heme group synthesis pathway, disturbances in the blood-brain barrier, and increase aquaporin expression have all been implicated, although there is a lack of evidence for a single or driving mechanism [18]. The fluorescence under 5-ALA may have an important clinical outcome as the complete resection of these lesions is usually curative [2], and therefore adjuvants to increase the extent of resection should be used.

From an imaging perspective, contrast enhancement in MRI and metabolic active lesion in PET correlate with 5-ALA fluorescence in lesions that are not typical for high-grade gliomas [8]. This was the rationale applied to operate this patient under 5-ALA guidance, and the fluorescence was expected.

Multiple reports can be found in the literature about high-grade glioma mimic surgery under 5-ALA guidance (inflammatory processes, radiation necrosis, multiple sclerosis, abscess and cerebral infarction) [13]. The surgical teams need to be aware of these lesions even though the natural history of high-grade gliomas (HGG) should be responsible for an aggressive surgical treatment regardless the literature reports HGG mimics with 5-ALA.

## Conclusion

WHO grade I papillary glioneuronal tumour may present as 5-ALA fluorescent lesions. From a clinical perspective, 5-ALA can be used to achieve complete resections in these lesions which, in most cases, can be curative.

## Electronic supplementary material

ESM 1. Resection of the tumour with 5-ALA. From second 20 onwards, shows fluorescene with Blue 400, showing the evidence for the use of Gliolan in low grade tumours. (MP4 15224 kb)
